# Supercritical CO_2_ Extraction of High-Added Value Compounds from *Chlorella vulgaris*: Experimental Design, Modelling and Optimization

**DOI:** 10.3390/molecules27185884

**Published:** 2022-09-10

**Authors:** Ioulia Georgiopoulou, Soultana Tzima, Vasiliki Louli, Kostis Magoulas

**Affiliations:** Laboratory of Thermodynamics and Transport Phenomena, School of Chemical Engineering, National Technical University of Athens (NTUA), Zografou Campus, 15780 Athens, Greece

**Keywords:** *Chlorella vulgaris*, bioactive compounds, phenolic content, chlorophylls, carotenoids, antioxidant activity, mass transfer, modelling, extraction optimization, cosolvent effect

## Abstract

Microalgae are well-known for their high-added value compounds and their recovery is currently of great interest. The aim of this work is the recovery of such components from *Chlorella vulgaris* through supercritical fluid extraction (SFE) with CO_2_. The effect of the extraction temperature (40–60 °C), pressure (110–250 bar), and solvent flow rate (20–40 g/min) was tested on yield, the extract’s antioxidant activity, and the phenolic, chlorophyll and carotenoid content. Thus, data analysis indicated that the yield was mainly affected by temperature, carotenoids by pressure, while the extract’s phenolics and antioxidant activity were affected by the synergy of temperature and pressure. Moreover, SFE’s kinetic study was performed and experimental data were correlated using Sovová’s mass transfer-based model. SFE optimization (60 °C, 250 bar, 40 g/min) led to 3.37% *w*/*w* yield, 44.35 mg_extr_/mg_DPPH_ antioxidant activity (IC50), 18.29 mg_GA_/g_extr_ total phenolic content, 35.55, 21.14 and 10.00 mg/g_extr_ total chlorophyll, carotenoid and selected carotenoid content (astaxanthin, lutein and β-carotene), respectively. A comparison of SFE with conventional aq. ethanol (90% *v*/*v*) extraction proved SFE’s superiority regarding extraction duration, carotenoids, antioxidant activity and organoleptic characteristics of color and odor despite the lower yield. Finally, cosolvent addition (ethanol 10% *w*/*w*) at optimum SFE conditions improved the extract’s antioxidant activity (19.46%) as well as yield (101.81%).

## 1. Introduction

Microalgae biomass presents remarkable commercial interest in the food, nutraceutical, cosmetic and pharmaceutical fields due to its high value-added bioactive components [1]. Τhe recovery of bioactive compounds with efficient and environmentally friendly technologies is an important subject of research. 

*Chlorella* is one of the most dominant microalgae genera in the global microalgae production that has already been industrialized [2,3]. *Chlorella vulgaris* (*C. vulgaris*), the most common *Chlorella* species, is a unicellular microorganism of a diameter not exceeding 10 μm with a wide application as a dietary supplement and food additive [4]. Along with several other microalgae genera, *Chlorella* is considered a source of bioactive natural substances such as chlorophylls, carotenoids and phenolic compounds [4,5,6]. However, the dark green color as well as the distinct fishy odor of *Chlorella* inhibits the wide exploitation in the demanding fields of food, dietary supplements and cosmetics [7,8]. The need to address this challenge has therefore arisen. 

Several studies have been conducted so far concerning the conventional *C. vulgaris* extraction for the recovery of bioactive compounds, including chlorophylls [9,10], carotenoids [9,10,11,12,13,14], and phenolic compounds [15], with various organic solvents [9,10,11,13]. The supercritical CO_2_ (scCO_2_) extraction of microalgae has also been studied in the framework of applying alternative methods with lower environmental impact. CO_2_ is considered an easily available, low-cost and safe solvent. Under supercritical conditions, CO_2_ presents improved dissolving power, while the extract’s thermal and chemical degradation are avoided [16]. However, studies of *C. vulgaris* supercritical fluid extraction (SFE) were mainly focused initially on lipid extraction [17,18,19,20,21,22,23,24] and then on carotenoids [10,12,13,14,19,20]. A limited number of published studies are related to other bioactive compounds, such as chlorophylls [10], and phenolic compounds or the extract’s bioactivity [25]. Moreover, the kinetic studies of the supercritical fluid extraction of *C. vulgaris* are also scarce [20,26,27].

The aim of this work is the extraction of bioactive and high-value added compounds from *Chlorella vulgaris* by studying the novel technique of supercritical fluid extraction with CO_2_. The work includes an integrated experimental study of SFE in a bench scale unit, process modelling, and optimization. To this end, the effect of three crucial operational parameters, namely extraction temperature, pressure and solvent flow rate, was investigated. The novelty of the study lies in the multifaceted evaluation of the method in terms of extraction yield and the extract’s antioxidant activity, phenolic, chlorophyll, and carotenoid content. Experimental data correlation of the examined responses was attempted, where possible, with proper statistical tools for the purpose of optimization. Additionally, representative conditions were selected for the correlation of experimental SFE yield data using the Sovová mass transfer model, under the assumptions of plug flow and the existence of phase equilibrium and diffusion controlled extraction regimes [28,29]. Moreover, a comparison of SFE with a conventional extraction technique was performed, resulting in SFE extract’s superiority in terms of bioactive content, antioxidant activity and market value. Finally, a SFE experiment with cosolvent addition was also performed, leading to enhanced yield and improved bioactive compound and antioxidant activity of the extract.

## 2. Results & Discussion

### 2.1. SFE of Bioactive Compounds

**Figure 1:**
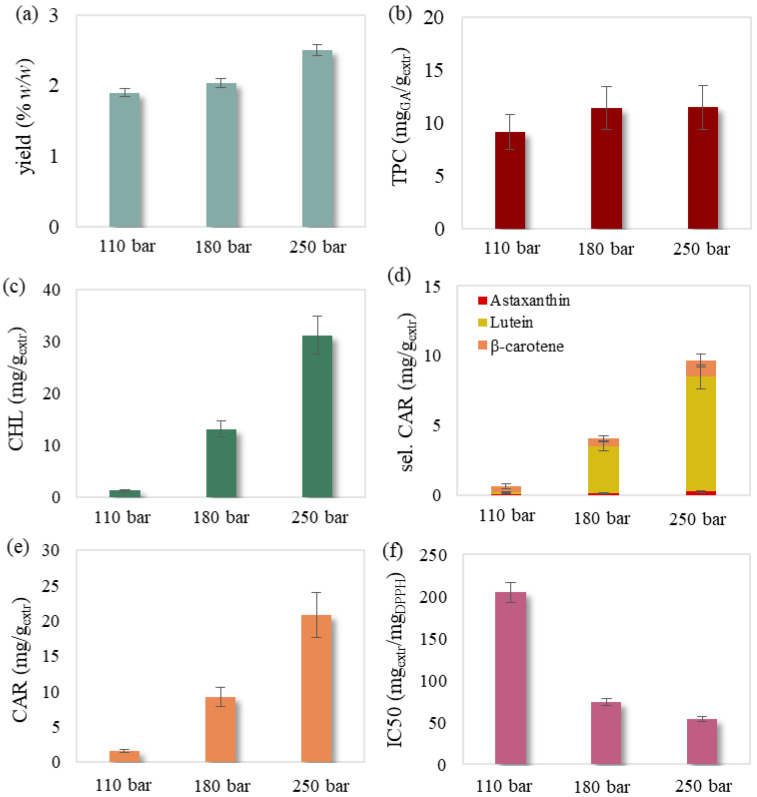
Pressure effect on SFE’s (**a**) yield, (**b**) total phenolic, (**c**) chlorophyll, (**d**) selected carotenoid, (**e**) total carotenoid content and (**f**) antioxidant activity examined at a temperature of 50 °C and a solvent flow rate of 30 g/min. The presented error bars express the standard deviation percentage of the central point replicates.

**Figure 2:**
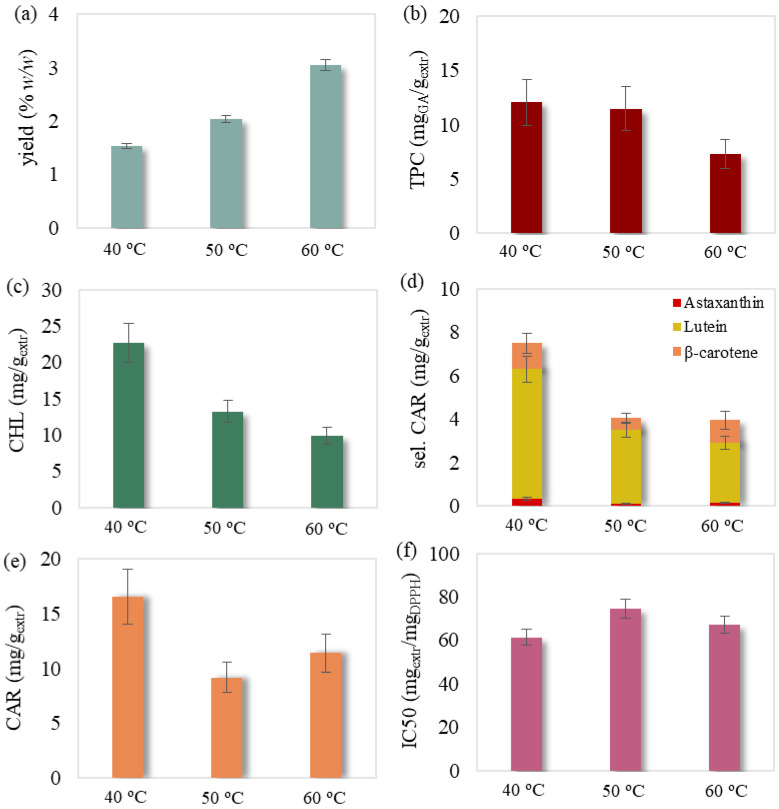
Temperature effect on SFE’s (**a**) yield, (**b**) total phenolic, (**c**) chlorophyll, (**d**) selected carotenoid, (**e**) total carotenoid content and (**f**) antioxidant activity examined at a pressure of 180 bar and a solvent flow rate of 30 g/min. The presented error bars express the standard deviation percentage of the central point replicates.

**Figure 3:**
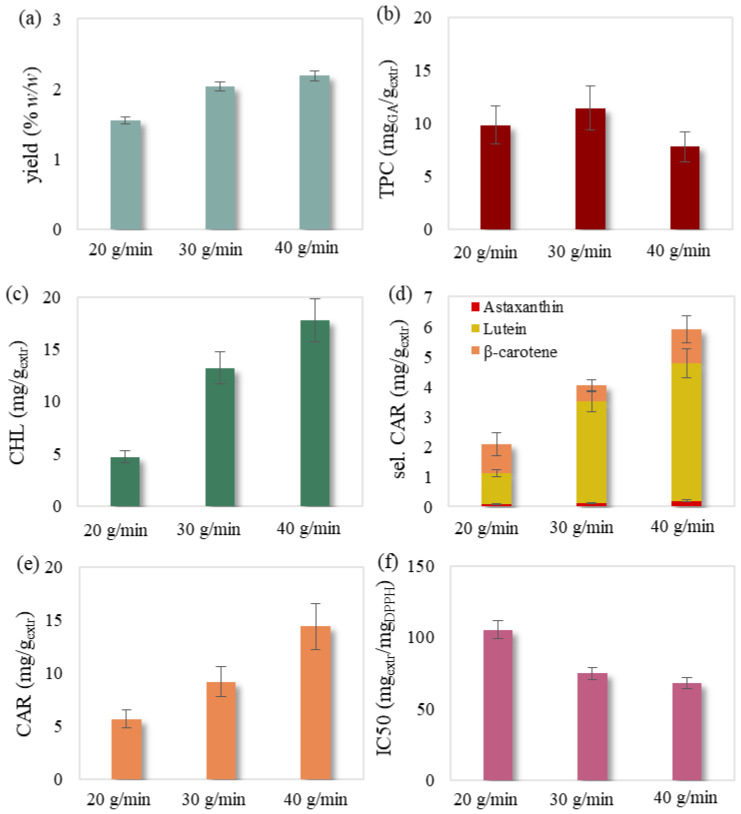
Flow rate effect on SFE’s (**a**) yield, (**b**) total phenolic, (**c**) chlorophyll, (**d**) selected carotenoid, (**e**) total carotenoid content and (**f**) antioxidant activity examined at a temperature of 50 °C and a pressure of 180 bar. The presented error bars express the standard deviation percentage of the central point replicates.

**Figure 4:**
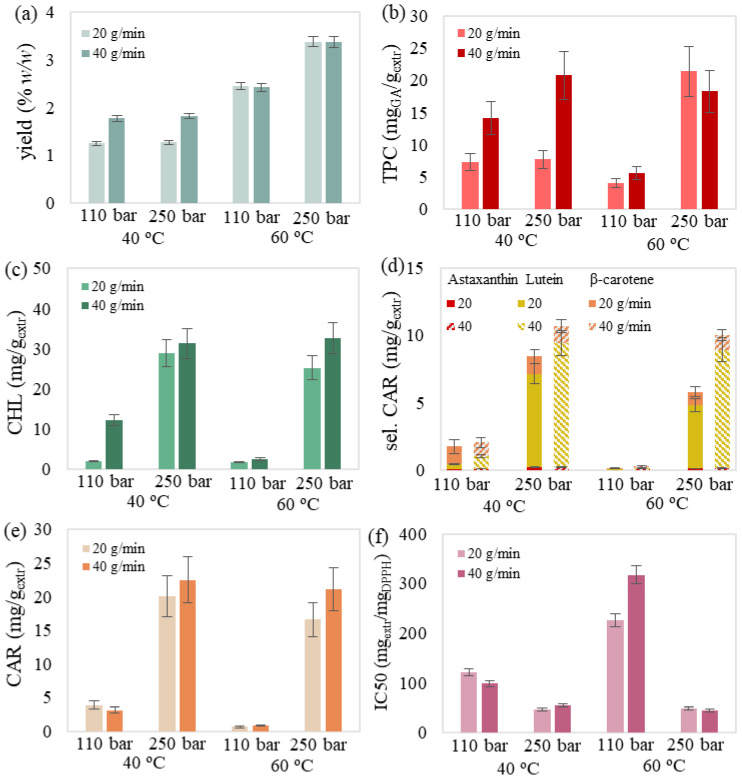
Simultaneous effect of temperature, pressure and flow rate on SFE’s (**a**) yield, (**b**) total phenolic, (**c**) chlorophyll, (**d**) selected carotenoid, (**e**) total carotenoid content and (**f**) antioxidant activity. The presented error bars express the standard deviation percentage of the central point replicates.

The obtained SFE extracts presented a dark yellow color and no fishy odor. The experimental results of the 18 experiments are presented in Table 1, while the examined effects of temperature, pressure and solvent flow rate are illustrated in Figure 1, Figure 2, Figure 3 and Figure 4.

Within the examined ranges of the operational conditions, the extraction yield varied from 1.25 to 3.38% *w*/*w*. These values are in accordance with extraction yields reported in the literature [18,30]. Carotenoid and chlorophyll content could also be considered comparable with those reported in other studies [10,14]. Reasonable value deviations are acceptable, taking into account the different cultivation, harvest and extraction conditions. 

It was also noted that selected carotenoids mainly consisted of lutein followed by significantly lower concentrations of β-carotene and astaxanthin (Figure 1d, Figure 2d, Figure 3d and Figure 4d). Regarding chlorophyll content, Jeffrey’s equations confirmed the presence of chlorophyll a. This could be attributed to the selectivity of relative non-polar supercritical CO_2_ towards the less polar chlorophyll a compared to the more polar chlorophylls b and c [31,32,33], which have been previously detected in this biomass [34].

#### 2.1.1. Effect of Pressure

The pressure effect during SFE was studied at 50 °C and 30 g/min. Pressure rise led to the significant increase of chlorophyll (Figure 1c) and carotenoid content (Figure 1d,e) as well as the remarkable improvement of the extract’s antioxidant activity (Figure 1f). The extraction of more polar carotenoids, such as lutein and astaxanthin [35], was justifiably enhanced by the pressure increase that also favored the polarity of supercritical CO_2_ [31]. A milder increase of extraction yield and phenolics was also noted (Figure 1c). Pressure rise under constant temperature was generally responsible for the density increase of CO_2_ and therefore its solvation capability [36]. The improved solvation power led to extracts richer in bioactive compounds and of higher yield. Such an observation was also made in other studies regarding the improved yield [10,13,20], as well as the increased carotenoid [10,13,20,37] and chlorophyll content [10] during pressure rise. 

#### 2.1.2. Effect of Temperature

In general, temperature increase under constant pressure during SFE is responsible for both decreasing the scCO_2_ density and increasing the vapor pressure of the extractable biomass compounds [38]. As a result, the solvent’s density drop decreases the solute’s solubility and thus does not favor SFE, while the rise of extractable compounds’ vapor pressure favors the extraction. In this study, the temperature effect on SFE was investigated at 180 bar and 30 g/min. Temperature elevation brought a remarkable increase in extraction yield (Figure 2a), and was also noted in the literature [20]. This is probably due to the dominating effect of vapor pressure increase of the extractable microalgae components in scCO_2_ at this certain pressure level. The obtained extract generally proved to be degraded in terms of phenolic (Figure 2b), chlorophyll (Figure 2c) and selected carotenoid content (Figure 2d), and was also observed for pigment content in other studies [13,21]. This could be attributed to the coextraction of other less- or non-bioactive substances at elevated temperatures [39]. On the other hand, total carotenoid content decreased abruptly from 40 to 50 °C and eventually increased at 60 °C (Figure 2e), a behavior also followed by antioxidant activity but with milder fluctuation (Figure 2f). The predominance of the vapor pressure effect of certain carotenoids while increasing temperature from 50 to 60 °C could be responsible for the increase of the total carotenoid content and consequently that of antioxidant activity. Such behavior for carotenoids is also reported in the literature [37].

#### 2.1.3. Effect of Solvent’s Flow Rate

The solvent’s flow rate effect was also noticeable in SFE at 50 °C and 180 bar (Figure 3). Despite the shortened residence time of scCO_2_ in the extraction vessel, flow rate rise provided an increased number of CO_2_ molecules in contact with the biomass [13]. The faster feed of pure solvent offered a greater concentration gradient and thus contributed to faster and enhanced diffusion phenomena. Therefore, flow rate increase presented a positive effect on the extract’s examined characteristics, also reported in the literature [13,40]. An exception occurred for the total phenolic content, which generally presented a rather noteworthy experimental error that did not allow safe conclusions (Figure 3b). It has been proven that extreme values of both low and high solvent flow rates do not favor SFE [41]. However, the chosen extraction conditions and flow rate range of this study might provide sufficient contact time between solvent and solute.

#### 2.1.4. Synergistic Effect

Determination of the combined effect of the examined variables was also attempted through Figure 4. The simultaneous increase of all three parameters led to higher SFE yield compared to the individual variable rise. However, the synergistic effect was considered more complex for the remaining responses, and limited conclusions were drawn. More specifically, phenolic (Figure 4b), chlorophyll (Figure 4c) and carotenoid content (Figure 4d,e), as well as the extract’s antioxidant activity (Figure 4f) were significantly favored at high pressures, regardless of the temperature and flow rate effect. The simultaneous increase of pressure and temperature also benefitted all responses. Finally, the increase of solvent flow rate generally caused either significant or mild improvement of the corresponding responses depending on temperature and pressure values, while any divergent results could be justified due to the experimental error.

The understanding of the synergistic effect of the independent variables is considered a complex and crucial issue that could be resolved through data correlation, the results of which are presented in the following section.

### 2.2. Statistical Analysis & Process Optimization 

#### 2.2.1. Regression & Reliability Analysis

A statistical analysis was performed by submitting the experimental data to ANOVA. Equations of 9 to 11 terms, including the intercept, were obtained according to Equations (8) and (9). The reduction of equation terms was attempted while maintaining each model’s hierarchy. During statistical analysis, total phenolic and chlorophyll content failed to correlate successfully and thus are not presented in the present work.

The regression model equations of yield (Equation (1)), antioxidant activity (Equations (2) and (3)), selected carotenoid content (Equations (4) and (5)) and total carotenoid content (Equations (6) and (7)) were expressed in real terms and are presented below.

(1) $$\begin{array}{ll} \mathrm{Yield}=3.0864 & -0.1758\;T-0.0225\;P+0.2032\;F+0.3223\;10^{-3}\;\mathrm{TP}+0.0023{\;T}^{2}+0.2786\;10^{-4}{\;P}^{2}\\ & -0.0019{\;F}^{2} \end{array}$$

(2) $$\begin{array}{ll} \mathrm{IC}50^{\prime }=-17.1444 & +0.9697\;T-0.0155\;P+0.5672\;F-0.3512\;10^{-3}\;T\;P-0.0240\;T\;F-0.0090{\;T}^{2}\\ & +0.6649\;10^{-4}{\;P}^{2}+0.2437\;10^{-3}{\;T}^{2}F \end{array}$$

(3) $$\mathrm{IC}50=\exp^{\mathrm{IC}50^{\prime }}$$

(4) $$\begin{array}{ll} \mathrm{sel}.{\;\mathrm{CAR}}^{\prime }=47.0266 & -1.8585\;T-0.1095\;P-0.8450\;F+0.0035\;T\;P+0.0420\;T\;F+0.0144{\;T}^{2}+0.2455{\;P}^{2}\\ & -0.0026{\;F}^{2}-0.4194{\;T}^{2}F+0.332210^{-5}{\;T\;P}^{2} \end{array}$$

(5) $$\mathrm{sel}.\;\mathrm{CAR}=\exp^{\mathrm{sel}.{\;\mathrm{CAR}}^{\prime }}$$

(6) $$\begin{array}{c} {\mathrm{CAR}}^{\prime }=36.5783-1.5855\;T+0.0423\;P-1.0244\;F+0.5022\;10^{-3}\;T\;P+0.0421\;T\;F+0.0143{\;T}^{2}-\\ 0.1373\;10^{-3}{\;P}^{2}-0.4129\;10^{-3}{\;T}^{2}F \end{array}$$

(7) $$\mathrm{CAR}=\exp^{{\mathrm{CAR}}^{\prime }}$$

The extraction yield is expressed in % *w*/*w*, antioxidant activity in mg_extr_/mg_DPPH_, total and selected carotenoid content in mg/g_extr_, while T, P and F stand for the extraction temperature (°C), pressure (bar) and solvent flow rate (g/min), respectively.

**Figure 5:**
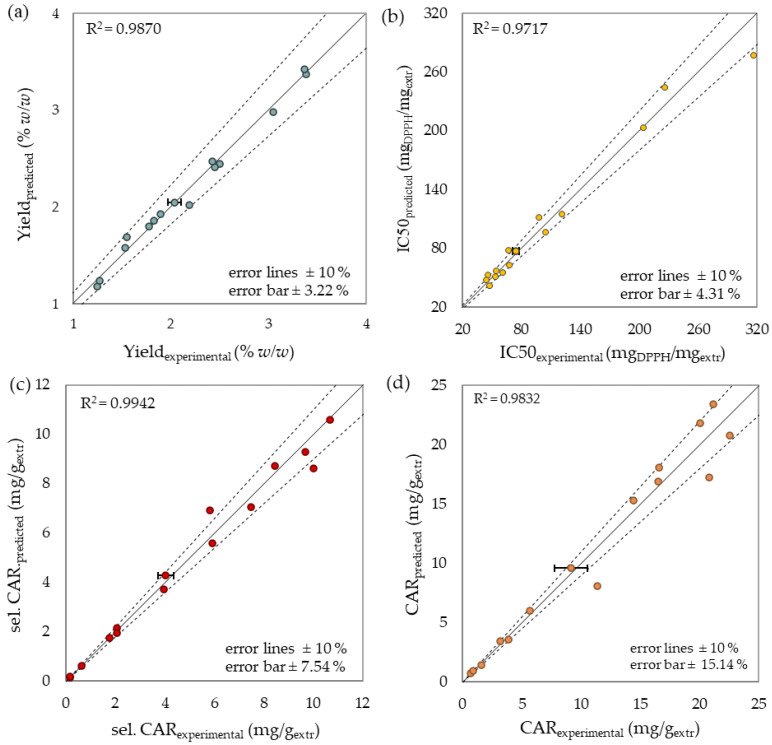
Experimental data versus predicted values of (**a**) yield, (**b**) antioxidant activity, (**c**) selected and (**d**) total carotenoid content. The experimental standard deviation was expressed using the error bars.

The main results of the performed reliability tests, including the F-test data of models, lack of fit (LOF), and significant equation terms, as well as adequacy measures, are presented in Table 2, and prove the sufficient correlation of the proposed models. In particular, F-tests’ *p*-values confirmed the statistical significance (*p* < 0.05) of the regression models and LOF’s insignificance (*p* > 0.1). The high values of coefficient of determination (R^2^) and high affinity between experimental and predicted data presented in Figure 5 also proved the models’ precision. Moreover, the fairly high values of adjusted R^2^ (Adj-R^2^) and the reasonable agreement with predicted R^2^ (Pred-R^2^) confirmed the reliable correlation. Models’ accuracy was justified by the desirable high values of adequate precision (Adeq Prec > 4). In contrast to all the other mentioned responses, total phenolic and chlorophyll content failed to correlate to an acceptable level and therefore were not included. Consequently, the proposed regression models could be considered handy for response estimation and prediction with relative high certainty.

Finally, *p*-value evaluation of the individual equation terms (Table 2) indicated the most statistically significant factors. Temperature was considered the most noteworthy factor for yield, and pressure was the most noteworthy factor for carotenoids, while the synergistic effect of temperature and pressure proved to be the most important factor in the case of antioxidant activity and total phenolic content. Solvent flow rate, although influential to some extent, seemed to be overshadowed by the other examined variables.

#### 2.2.2. Response Surface Plots

**Figure 6:**
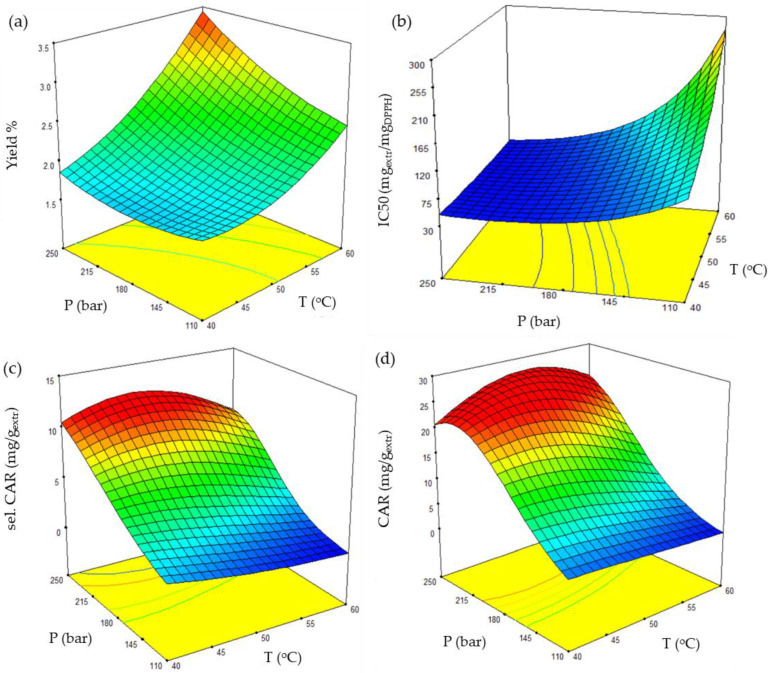
3-D plots presenting the combined effect of pressure and temperature on (**a**) yield, (**b**) antioxidant activity, (**c**) selected and (**d**) total carotenoid content. Solvent flow rate is set at 40 g/min.

A comprehensive perception of variable interaction and thus SFE optimization was achieved through surface plots of the successfully examined response models, as shown in Figure 6. According to Table 2, pressure and temperature were found to be the most significant terms, either individually or combined, and therefore were common axial terms for all graphs.

The significant variation presented in the graphs of Figure 6 confirmed the noteworthy effect of temperature and pressure on the *C. vulgaris* SFE of bioactive compounds. The maximized yield was estimated at 60 °C and 250 bar. Any temperature or pressure decrease resulted in an intense and milder yield reduction, respectively, while their simultaneous decrease led to a more pronounced drop in yield (Figure 6a).

The most attenuated antioxidant activity derived from SFE was estimated at 60 °C and 110 bar. The temperature decrease and pressure increase appeared to improve the extract’s bioactivity. However, the temperature effect proved less important than pressure, and optimal antioxidant activity was estimated at higher pressures (Figure 6b).

Carotenoid composition was consistent with the extract’s antioxidant activity. Both selected and total carotenoids presented similar response surfaces, and maximum carotenoid content was estimated at 250 bar (Figure 6c,d).

#### 2.2.3. SFE Parameter Optimization

One of the main aims of this work was the optimization of the examined operational conditions of *C. vulgaris* SFE. According to Design Expert^®^, a proposed set of operating conditions for simultaneous maximization of extraction yield, the extract’s carotenoid content (total & selected), as well as the antioxidant activity were 59.12 °C, 250 bar and 36.32 g/min. These values are considered similar to the experimentally optimal observed ones (60 °C, 250 bar and 40 g/min), which are thus proposed as the final optimal conditions in the examined range of extraction temperature, pressure and solvent flow rate.

### 2.3. Kinetic Modelling of SFE

**Figure 7:**
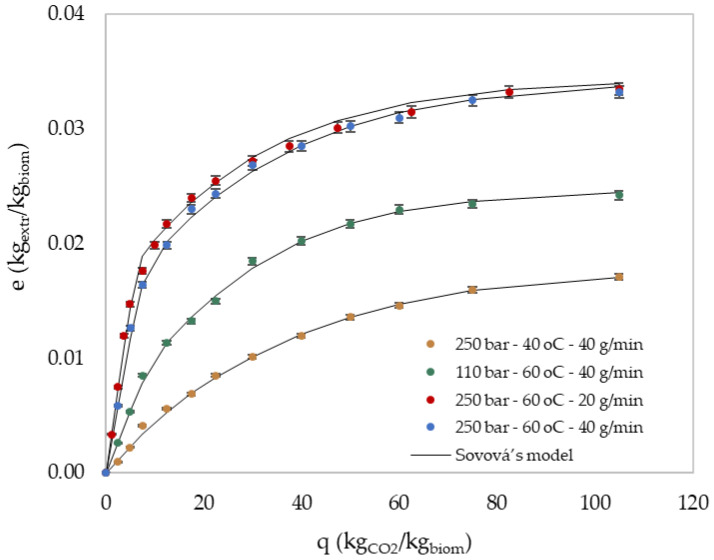
Effect of temperature (blue & yellow), pressure (blue & green), and solvent flow rate (blue & red) on yield (kg_extr_/kg_biom_) versus specific amount of solvent (kg_CO2_/kg_biom_). Correlation results with Sovová’s model are also included. The experimental standard deviation was expressed using the error bars.

The proposed Sovová model [28,29] was successfully applied to the experimental SFE data of representative operational conditions, as shown in Figure 7 and Table 3, where the estimated model parameters are also presented. According to Figure 7, model curves sufficiently coincided with the experimental data. Additionally, the absolute average deviation (AAD%) for each data set remained low, as shown in Table 3. 

The extracts’ solubility ($y_{r}$) and the concentration of the initial solute in the solid phase ($x_{0}$) varied as a function of the extraction’s temperature and pressure as a consequence of CO_2_ density and solute vapor pressure variation, which is also reported in other studies [42,43]. Both temperature and pressure rise led to the increase of $y_{r}$ and $x_{0}$. Like $x_{0}$, $x_{k}$ followed a slightly upward trend, with a temperature and pressure increase. During the solvent flow rate effect study, the values of $y_{r}\;$ and $x_{k}$, which are highly dependent on the state of the fluid and therefore on the pressure and temperature [44], remained rightly intact. The solvent flow rate decrease caused a slight upward displacement of the extraction curve (Figure 7), possibly due to the longer residency of the solvent inside the extractor and the longer contact time with the biomass. However, it did not noticeably affect the overall extraction procedure, leading to a similar extraction yield at 250 bar.

Regarding the mass transfer parameters, Z$\dot{q}$ variation did not significantly affect the process description, and the limited experimental data of the first extraction stage pointed out the risk of uncertain calculation. Therefore, a common Z$\dot{q}$ value was suitably adapted for all experiments except for SFE-60 °C-110 bar-40 g/min, where the pressure drop led in Z$\dot{q}$ increase.

On the other hand, different W$\dot{q}$ values were obtained during the variation of operational conditions and reflected the SFE rate changes. In particular, the W$\dot{q}$ parameter was mainly affected by solvent flow proportionally, indicating that the third extraction stage could be affected by the external mass transfer, a phenomenon also observed in other studies [45,46]. Temperature and pressure affected W$\dot{q}$ less in an inversely proportional way, but due to the variation of *x_0_*, no safe conclusions can be made. In general, Z$\dot{q}$ prevailed over W$\dot{q}$ with a two-order magnitude deviation, which was also observed in other studies [46]. Lower W$\dot{q}$ values indicated greater resistance in the solid phase, which affects the slower extraction stage. 

### 2.4. SFE versus SLE

The two methods were performed under the optimal conditions presented in Table 4. The proposed conventional extraction method was considered a simple process with lower fixed costs. However, in contrast to SFE, SLE required an additional separating step of solvent-solute (vacuum evaporation) and was extremely time-consuming (more than seven times longer). 

**Figure 8:**
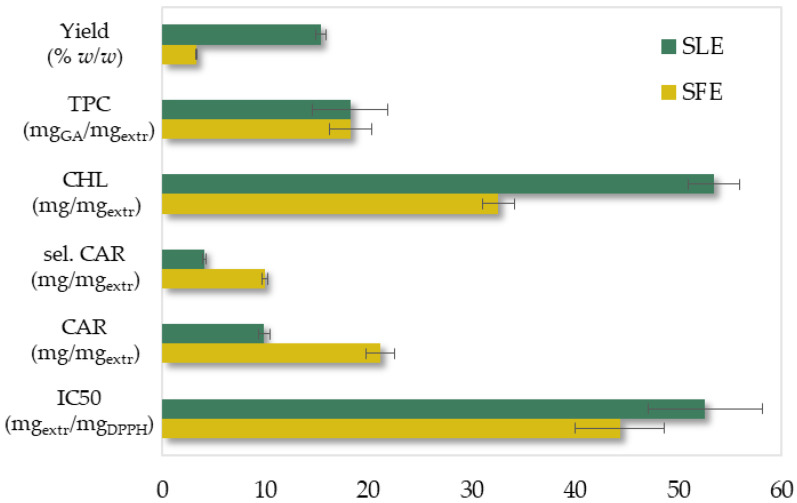
The examined responses of SLE and SFE extracts under the optimal extraction conditions.

According to the experimental results presented in Figure 8, the SFE resulted in a 4.6 times lower yield with a slightly improved extract antioxidant activity, similar phenolic content and 1.6 times less chlorophylls. Despite the fact that the phenolic and chlorophyll content has not been successfully correlated and was not included during SFE optimization, they were satisfactorily recovered under those operational conditions. Finally, the SFE extract was more than twice enriched in selected and total carotenoids. The SFE extract’s superiority in carotenoids, which are high value-added components (250–2000 USD/kg) [47], significantly increases its value and makes it more commercially attractive.

Moreover, the received SLE extract presented a dark green color and a slight fishy odor in contrast with the dark yellow extract with no fishy smell of SFE. SLE favored chlorophyll extraction and therefore led to a green extract, whereas SFE’s higher selectivity towards carotenoids led to a dark yellow extract. Furthermore, the fishy smell of microalgae is usually due to the presence of volatile organic compounds—VOCs [48]. SLE probably favored the extraction of those volatile compounds while the abrupt depressurization during SFE could have led to the loss of those extracted volatiles [49]. The aforementioned characteristics could facilitate the wide exploitation of the SFE’s extract in the demanding fields of food, dietary supplements and cosmetics.

In conclusion, SFE proved to be an attractive alternative to SLE in terms of extract quality and time-consumption.

### 2.5. Effect of Cosolvent

**Figure 9:**
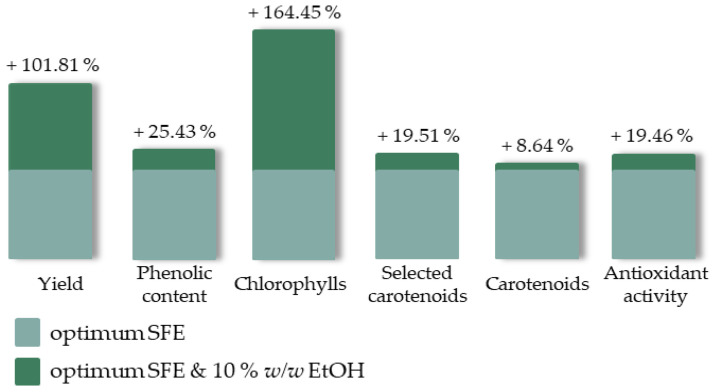
The effect of cosolvent addition during SFE on the examined responses expressed as a percentage alteration.

SFE is usually enhanced by the addition of different cosolvents. Ethanol is considered a green solvent, safe for food processing and human consumption/use. It is commonly used in SFE and typically low concentrations are employed in order to enhance the extraction’s efficiency [50,51]. Ethanol, 10% *w*/*w*, was added during SFE under the optimum extraction conditions and the cosolvent’s effect is shown in Figure 9. The corresponding responses of the reference SFE experiment (see Table 1-SFE-17) are presented in a common basis with light green, while the percentage change due to the cosolvent addition is presented in dark green and is also indicated above each column. Indeed, the cosolvent addition improved all the examined responses, especially the extraction yield and the extract’s chlorophyll content. The solvent’s polarity change contributed to the extraction of more polar compounds, such as phenolic components (phenolic compound recovery accomplished with both polar and non-polar solvents [52]), chlorophylls (polar aldehyde group is found in chlorophyll b molecules [53]) and carotenoids (xanthophylls are considered more polar than carotenes [54]) and, as already established [51], favored extract’s antioxidant activity. Finally, the obtained extract presented a yellowish green color and no particular fishy odor.

## 3. Materials and Methods

### 3.1. Materials

Commercial *C. vulgaris* biomass originated from South China was purchased from Go Superfoods Ltd. (Sheffield, UK). Cultivation was performed in natural water open ponds and mesh screens were used for biomass harvesting. Milling was used as a pretreatment method and powder form biomass occurred after spray drying. All the referred stages of biomass production were in accordance with strict regulations for human consumption-intended products. The primary composition of *C. vulgaris* biomass was determined in previous work [34] and is presented in Table 5.

Carbon dioxide (99.5 %) was purchased from Air-Liquide Hellas (Athens, Greece). Ethyl acetate, orthophosphoric acid (analytical grade reagents), methanol (≥99.8%), ethanol (absolute, HPLC grade) tert-butyl-methyl ether (MTBE), water (HPLC grade reagents), and anhydrous sodium carbonate (99.5%) were purchased from Fisher Scientific International Inc. (Pittsburgh, PA, USA). Standard compounds of astaxanthin (≥98%), lutein (≥92%), and β-carotene (≥95%) for HPLC analysis were purchased from Acros Organics BVBA (Antwerp, Belgium), Extrasynthese SAS (Lyon, France), and Sigma Aldrich Co. (Saint Louis, MO, USA), respectively. Gallic acid (98%) (ACS reagents) was purchased from Acros Organics BVBA (Antwerp, Belgium). Free radical 2,2-diphenyl-1-picrylhydrazyl (DPPH) and Folin-Ciocalteu reagent were purchased from Sigma Aldrich Co. (Saint Louis, MO, USA) and Carlo Erba Reagents SAS (Milan, Italy), respectively. 

### 3.2. Biomass Extraction

#### 3.2.1. Supercritical Fluid Extraction (SFE) with CO_2_

Supercritical CO_2_ extraction was performed in a bench scale apparatus (SFE-500, SEPAREX CHIMIE FINE, Champigneulles, France). The detailed apparatus description and extraction procedure is described in previously published work [55].

In this work, 80 g of *C. vulgaris* biomass were loaded in the extractor vessel. Dead space reduction and uniform flow distribution were achieved with the addition of glass bead (d = 4.5 mm) layers at the top and bottom of the vessel. The two separators operated at 60 and 10 bar respectively and 8 °C. Additionally, preliminary experiments showed that exhaustive extraction of *C. vulgaris* biomass was achieved with 94 kg_CO2_/kg_biom_, and therefore the solvent consumption was set at 100 kg_CO2_/kg_biom_ for all of the performed experiments. The operational conditions of pressure, temperature and solvent flow rate were adjusted according to an experimental design and experimental error was determined through a quadruplicate repetition of the central point (see Table 1). The yield was determined by total biomass weight loss of the extraction vessel at the end of each experiment and all collected extracts were stored at −18 °C until further analysis.

In the case of the kinetic study, experiments were interrupted at regular periods of time for weight loss measurement. The experimental error was calculated from duplicate experiments. 

Regarding the cosolvent addition, ethanol was inserted through a piston pump and ethanol content in CO_2_ was set to 10% *w*/*w*. 

#### 3.2.2. Solid-Liquid Extraction (SLE) with aq. Ethanol 90% *v*/*v*

Conventional extraction was performed with 1 g of *C. vulgaris* biomass and 37 mL of aq. ethanol 90% *v*/*v*. Sample and solvent were loaded into a jacketed vessel, stirred at 500 rpm and heated at 30 °C for 24 h in the dark by using a Carousel tech stirring hotplate (Radleys, Essex, UK). A condenser was connected to the top of the vessel for the minimization of solvent losses. The proposed solid-liquid extraction conditions have been optimized in a previously published study [34]. After extraction, the mixture was centrifuged for 8 min at 3000 rpm using a Hermle centrifuge Z206-A (Hermle AG, Baden-Württemberg, Germany). The supernatant was filtered using a ChromPure PTFE/L 0.45 μm filter (Membrane solutions, LLC, North Bend, OH, USA) and vacuum evaporated at 45 °C and 100 mbar using a Hei-VAP Advantage ML rotary evaporator (Heidolph Instruments GmbH & Co. KG, Bayern, Germany). SLE was performed in duplicate and the dry microalgal extracts were obtained after evaporation and stored at −18 °C until further analysis. 

### 3.3. Extract Characterization

All extracts were characterized in terms of their bioactivity, phenolic, chlorophyll and carotenoid content, as well as concentration in selected carotenoids of great interest, i.e., astaxanthin, lutein and β-carotene. 

#### 3.3.1. Spectrophotometric Assays

The DPPH free radical scavenging assay, as described by Laina et al. [56], was applied for the antioxidant activity determination. The half-maximal inhibitory concentration (IC50) was detected at 515 nm and expressed in mass ratio of extract to DPPH (mg_extr_/mg_DPPH_). 

Determination of the phenolic compounds was performed through the Folin–Ciocalteu assay, as described by Drosou et al. [57]. The total phenolic content (TPC) was detected at 765 nm and expressed in mass ratio of gallic acid equivalent to extract (mg_GA_/g_extr_). 

Furthermore, total carotenoid (CAR) and chlorophyll (CHL) quantification was performed through equations provided by Jeffrey et al. [58,59]. The determination of CAR and CHL included absorbance measurements at 480, 510, 630, 647 and 664 nm, and were expressed in mass ratio of the corresponding compound to extract (mg/g_extr_). All required spectrophotometric measurements were performed in a Shimadzu UV-1900i UV–Vis Spectrophotometer (Shimadzu Corporation, Kyoto, Japan) using 1 cm length quartz cuvettes.

#### 3.3.2. Reversed-Phase-High Performance Liquid Chromatography (RP-HPLC)

Carotenoid separation and determination of the selected carotenoids of astaxanthin, lutein and β-carotene were conducted by performing the RP-HPLC analysis. Carotenoid separation was achieved by performing the reported gradient procedure of Stramarkou et al. [60] and the selected carotenoid content (sel. CAR) was then expressed in mass ratio of the astaxanthin, lutein and β-carotene sum to the extract (mg/g_extr_). The RP-HPLC analysis system consisted of a Jasco LG-1580-04 gradient unit, a Jasco PU-1580 HPLC pump (Jasco Inc., Easton, MD, USA), a Rheodyne 7125 injector (Rheodyne Europe GmbH, Bensheim, Germany) with 20 μL loop, a Jones 7955 column chromatography heater (Jones Chromatography Limited, Wales, UK) and a Shimadzu SDP-M20A Diode Array Detector (DAD) (Shimadzu Corporation, Kyoto, Japan). 

All of the assays performed for the extract characterization are described in detail in previous publication [34].

### 3.4. Experimental Design, Statistical Analysis and Optimization

The experimental design was determined by the Response Surface Methodology (RSM) of Face-Centered Central Composite Design (FC-CCD). The effect of three independent variables, namely, extraction temperature (T), pressure (P) and solvent flow rate (F), was studied according to the six individual responses of yield, antioxidant activity (IC50), total phenolic (TPC), chlorophyll (CHL) and carotenoid (CAR) content, as well as selected carotenoid content (sel. CAR), i.e., astaxanthin, lutein and β-carotene. The experimental design considered three different levels of each variable (−1, 0, +1) and included axial (6), factorial (8) and central (4 repetitions) points. The examined range of the independent variables of extraction temperature, pressure and solvent flow rate were 40–60 °C, 110–250 bar and 20–40 g/min, respectively. All the setpoint combinations of the experimental design in terms of real variables are presented in Table 2.

Eventually, an analysis of variance (ANOVA) was performed for the assessment of the experimental data through Equation (1), while response transformation was also applied, where deemed necessary, according to Equation (2).

(8) $$Y=b_{0}+\overset{i=1}{\underset{3}{\sum }}b_{i}X_{i}+\overset{i=1}{\underset{3}{\sum }}b_{\mathrm{ii}}X_{i}^{2}+\overset{i=1}{\underset{2}{\sum }}\overset{j=i+1}{\underset{3}{\sum }}b_{\mathrm{ij}}X_{i}X_{j}+\overset{i=1}{\underset{2}{\sum }}\overset{j=i+1}{\underset{3}{\sum }}b_{1\mathrm{ij}}X_{i}X_{j}^{2}+\overset{i=1}{\underset{2}{\sum }}\overset{j=i+1}{\underset{3}{\sum }}b_{2\mathrm{ij}}X_{i}^{2}X_{j}$$

(9) $$\;Y^{\prime }=f\left(Y\right)↔Y=f\left(\;Y^{\prime }\right)$$

where, Y and Y′ represent the corresponding examined response and transformation, b_0_ the mean and b_i_, b_ii_, b_ij_, b_1ij_ and b_2ij_ stand for the coefficients, and X_i_ and X_j_ are the chosen independent variables of extraction temperature, pressure and solvent flow rate, respectively.

Statistical significance was determined through the Fisher’s statistical test (F-test) with 95% significance level. The Design Expert^®^ Version 13 software trial (Stat-Ease Inc., Minneapolis, MN, USA) was used for the experimental design, modelling and statistical analysis of the experimental data.

### 3.5. Mathematical Model of Extraction Kinetics

The mass balance model proposed and further developed by Sovová and coworkers [28,29] was employed for the correlation of the experimental data of supercritical fluid extraction, as it is considered suitable for the description of supercritical fluid extraction of natural compounds from microalgae [26].

It is based on an extended version of Lack’s plug flow model [61]. Briefly, the extraction process is divided into three stages. The first one (I) includes the extraction of the easily accessible compounds and is considered the fast stage, with a constant extraction rate. The last one (III) involves the diffusion-controlled stage of the slow compound extraction from the inside of the substrate particles. Finally, the transition from the fast to the slow extraction stage is expressed by an intermediate one (II). 

The equations of the model are presented below, while all of the assumptions and development details of the model are extensively presented elsewhere [28,29].

(10) $$e=\{\begin{array}{c} q\;y_{r}\;\left[1-exp^{-Z}\right],\;q<q_{m}\;\left(I\right)\\ y_{r}\;\left[q-q_{m}\exp\left(z_{W}-Z\right)\right],\;q_{m}<q<q_{n}\;\left(II\right)\\ x_{0}-y_{r}/W\;ln\{1+\left[exp\left(W\;x_{0}/y_{r}\right)-1\right]\;exp\left[W\left(q_{m}-q\right)\right]\;x_{k}/x_{0}\},\;q\geq q_{n}\;\left(III\right)\; \end{array}$$

The additional required values are determined using the following equations:

(11) $$q_{m}=\left(x_{0}-x_{k}\right)/y_{r}\;Z$$

(12) $$q_{n}=q_{m}/W\;ln\left[x_{k}+\left(x_{0}-x_{k}\right)\;exp\left(W\;x_{0}/y_{r}\right)\right]\;$$

(13) $$z_{w}/Z=y_{r}/\left(W\;x_{0}\right)\;ln\left(x_{0}\;exp\left[W\;\left(q-q_{m}\right)\right]-x_{k}\right)/\left(x_{0}-x_{k}\right)\;$$

(14) $$Z=k_{f}\;\alpha_{0}\rho /\dot{q}\;\left(1-\varepsilon \right)\;\rho_{s}\;$$

(15) $$W=k_{s}\;\alpha_{0}/\dot{q}\;\left(1-\varepsilon \right)\;$$

where, e represents the specific amount of the extracted solute (kg_extr_/kg_solute-free feed_), q represents the specific amount of the passing solvent through the extractor (kg_solv_/kg_solute-free feed_), $q_{n}$ is the specific amount of the passing solvent when the easily accessible solute is totally extracted and $q_{m}$ represents the specific amount of the passing solvent when the extraction inside the particles begins. Following, $y_{r}$ represents the solute’s solubility in the solvent (kg_solute_/kg_solv_), $x_{0}$ represents the initial concentration of the solute in the solid phase (kg_solute_/kg_solute-free feed_), $x_{k}$ represents the concentration of the less accessible solute in the solid (kg_solute_/kg_solute-free feed_), $z_{w}$ is the dimensionless coordinate between slow and fast extraction and $k_{s}$ and $k_{f}$ represent the mass transfer coefficient of solid and solvent-phase respectively (m/s). Finally, Z and W represent the dimensionless mass transfer parameters in the fluid and solid phase respectively, ρ and $\rho_{s}$ is the density of the solid and solvent’s density (kg/m^3^) correspondingly, $\alpha_{0}$ is the specific interfacial area (m^2^/m^3^), $\dot{q}$ is the specific flow rate (s^−1^) and ε is the bed void fraction.

Regarding the independent parameters of the model, *x_0_* could be considered equal to the experimental value resulted from the exhaustive extraction under the corresponding experimental conditions. Moreover, $y_{r}$ could be estimated from the curve slope during the first extraction stage (I). Finally, the variables $x_{k}$, Z and W were fitted by minimizing the absolute average deviation (AAD) of *e* as objective function given by Equation (16). The fitting parameters were subjected to the following constraints: 0<Z*,* W<10 and $0\;\leq \;x_{k}\;\leq \;x_{0}$.

(16) $$\mathrm{AAD}\;\left(\%\right)=\frac{100}{N}\;\overset{N}{\underset{i=1}{\sum }}\frac{\left|e_{i}^{predicted}-e_{i}^{experimental}\right|}{e_{i}^{experimental}}\;$$

where, N stands for the number of experimental points of each experiment.

## 4. Conclusions

In the current work, the temperature, pressure and solvent flow rate’s effect on the recovery of bioactive *C. vulgaris* extracts through supercritical fluid extraction with CO_2_ was examined. The study included the determination of the extraction yield as well as extract analysis in terms of phenolic, chlorophyll and carotenoid content, and antioxidant activity. The employment of FC-CCD contributed to the construction of the experimental design and the ANOVA assisted in the study of the effect of the selected variables. Data correlation led to reliable models for all the examined responses except total chlorophyll and phenolic content.

A statistical analysis proved that the most significant effect on extraction yield was temperature variation, while pressure highly affected carotenoid content and the combined temperature-pressure term decisively influenced the extract’s antioxidant activity and phenolic content.

Consequently, process optimization indicated that 60 °C, 250 bar and 40 g/min were the optimal conditions of temperature, pressure and solvent flow rate for maximum extract recovery with improved antioxidant activity and superiority in carotenoid content, as well as the satisfactory presence of chlorophylls and phenolics.

Additionally, representative conditions were selected for the kinetic study of SFE. Sovová’s model successfully described the SFE curves and proved its applicability in the *Chlorella vulgaris* biomass. The results could be useful for process simulation, scale-up and further optimization; thus, further study and determination of characteristic sizes of the extraction bed and biomass are required for more accurate results.

Furthermore, a comparison of SFE with conventional extraction with aq. ethanol concluded that in contrast to the quite efficient SLE, the faster and carotenoid-selective SFE provided commercially valuable extracts with higher antioxidant activity and improved color and odor characteristics.

Finally, the cosolvent addition showed that ethanol presence, 10% *w*/*w*, increased the bioactive compound content, improved the extract’s antioxidant activity, and contributed to addressing the low SFE efficiency issue.

## Figures and Tables

**Table 1:** The experimental responses of yield, antioxidant activity (IC50), total phenolic (TPC), chlorophyll (CHL), selected carotenoid (sel. CAR) and carotenoid (CAR) content.

Run	T (°C)	P (bar)	Flow (g/min)	Time (h)	Yield (% *w*/*w*)	IC50 (mg_extr_/ mg_DPPH_)	TPC (mg_GA_/g_extr_)	CHL (mg/g_extr_)	Sel. CAR (mg/g_extr_)	CAR (mg/g_extr_)
SFE-1	40	180	30	4.44	1.54	61.36	12.04	22.71	7.48	16.52
SFE-2	40	110	20	6.67	1.25	121.64	7.27	1.90	1.76	3.91
SFE-3	40	110	40	3.33	1.77	97.97	14.13	12.08	2.05	3.15
SFE-4	40	250	20	6.67	1.27	46.48	7.76	28.86	8.44	20.04
SFE-5	40	250	40	3.33	1.83	54.27	20.76	31.26	10.69	22.51
SFE-6	50	110	30	4.44	1.90	204.83	9.17	1.33	0.65	1.59
SFE-7	50	180	20	6.67	1.55	105.36	9.87	4.71	2.08	5.67
SFE-8	50	180	30	4.44	2.11	80.43	12.50	12.08	4.55	8.30
SFE-9	50	180	30	4.44	1.96	74.76	10.11	11.37	3.77	7.43
SFE-10	50	180	30	4.44	1.99	68.25	14.26	14.25	385	10.03
SFE-11	50	180	30	4.44	2.10	75.01	9.01	15.16	3.98	10.96
SFE-12	50	180	40	3.33	2.19	68.03	7.81	17.79	5.91	14.41
SFE-13	50	250	30	4.44	2.50	54.38	11.51	31.23	9.68	20.81
SFE-14	60	110	20	6.67	2.45	226.12	4.06	1.66	0.17	0.66
SFE-15	60	110	40	3.33	2.42	317.03	5.57	2.44	0.15	0.87
SFE-16	60	250	20	6.67	3.38	47.88	21.38	25.18	5.82	16.59
SFE-17	60	250	40	3.33	3.37	44.35	18.29	32.55	10.00	21.14
SFE-18	60	180	30	4.44	3.05	67.30	7.30	9.89	3.96	11.40
SD * (%)					±3.22	±5.78	±17.84	±11.68	±7.54	±15.14

* Standard deviation of the central point experiments (Runs: SFE-8 to SFE-11).

**Table 2:** The main ANOVA results and adequacy measures of the responses examined for the SFE of *C. vulgaris*.

Yield		IC50		Sel. CAR.		CAR	
Source	*p*-Value	Source	*p*-Value	Source	*p*-Value	Source	*p*-Value
Model	<0.0001	Model	<0.0001	Model	<0.0001	Model	<0.0001
T	<0.0001	P	0.005	P	0.0001	T	0.0236
P	0.0003	F	0.0426	F	0.0019	P	0.0013
F	0.0005	TP	0.0005	P^2^	0.0004	F	0.0084
TP	0.0001	T^2^	0.0479	F^2^	0.0276	TP	0.0007
TF	0.003	P^2^	0.0018	T^2^F	0.0104	P^2^	0.0002
T^2^	0.004	T^2^F	0.0432	TP^2^	0.0208	T^2^F	0.0259
P^2^	0.05						
F^2^	0.01						
LOF	0.296		0.1025		0.1080		0.4358
R^2^	0.987		0.9717		0.9941		0.9832
Adj-R^2^	0.976		0.9466		0.9858		0.9683
Pred-R^2^	0.944		0.7934		0.8934		0.9372
Adeq Prec	32.11		20.52		35.97		25.12

**Table 3:** Optimal estimated parameter values of the Sovová model for the *C. vulgaris* SFE.

P (bar)	T (°C)	F (g/min)	*y_r_*	*x*_0_	*x_k_*	$Z\dot{q}$ 10^2^ (s^−1^)	$W\dot{q}$ 10^4^ (s^−1^)	AAD * (%)
250	40	40	0.0006	0.018	0.0132	1.18	3.02	3.29
110	60	40	0.0012	0.025	0.0167	2.38	3.83	2.04
250	60	20	0.0032	0.036	0.0175	1.18	1.58	3.03
250	60	40	0.0032	0.036	0.0175	1.18	2.86	1.66

* absolute average deviation of the experimental from the predicted points (see Equation (16)).

**Table 4:** Optimal conditions of SLE and SFE.

Parameter	SLE [34]	SFE
solvent	EtOH 90% *v*/*v*	CO_2_
solvent-to-biomass (kg/kg_biom_)	30	100
stirring (rpm)	500	n/a *
T (°C)	30	60
P (bar)	1	250
F (g/min)	n/a *	40
Duration (h)	24	3.3

* not applicable.

**Table 5:** Primary composition of the commercial *C.vulgaris* biomass as reproduced from Georgiopoulou et al. [34] licensed under CC BY 4.0.

Primary Composition	% *
Lipid	22.17 ± 0.46
Carbohydrate	33.84 ± 1.33
Protein	44.48 ± 0.77
Ash	5.63 ± 0.06
Moisture	2.32 ± 0.12

* All values except moisture are expressed on dry basis (dw).

## Data Availability

Additional data for this study are not available on public database; the corresponding author can provide them upon request.
